# Drinking Water Quality: Lead Particles on Tap

**DOI:** 10.1289/ehp.116-a201

**Published:** 2008-05

**Authors:** Rebecca Renner

Lead exposure is a serious concern for children’s health. Lead impairs children’s brain development, and many scientists believe no dose is safe. High levels of lead in the paint used on children’s toys resulted in numerous toy recalls in 2007. But some school drinking water may also expose children to high levels of lead, according to environmental engineer Marc Edwards of Virginia Polytechnic Institute and State University.

Children’s toys and jewelry are recalled if the accessible lead dose—the amount expected to actually make its way into a child—exceeds 175 μg. In an eight-ounce glass of water, 175 μg lead converts to 700 ppb, according to Edwards, who notes that levels this high have been reported in school drinking fountains in Washington, DC; Seattle, Washington; and communities in Virginia and Massachusetts.

In 2007 more than 10% of the Washington, DC, schools Edwards tested had at least one fountain with lead levels greater than 700 ppb. He says the most contaminated sample contained 20,000 ppb lead, equivalent to eating 14 dime-sized pieces of paint containing more than 16 times the Consumer Product Safety Commission limit for lead in paint. Some of this lead is dissolved in the water, but much of it remains in particulate form, originating primarily from old lead solder. Edwards presented these findings at “20 Years of Success and a Vision for the Future,” a December 2007 meeting cosponsored by the NIEHS Superfund Basic Research Program.

Elevated blood lead levels in children from North Carolina and Washington, DC, have been traced by local public health authorities back to particles of solder in drinking water. In each case a change in water treatment appears to have exacerbated corrosion of water pipes, releasing more particles from solder into the water. Water filters that are attached to the faucet remove both dissolved and particulate lead, but pitcher-type filters do not, according to NSF International, which certifies water filters.

Scientists have considered solder to be a minor source of lead in drinking water because they believed that, over time, mineral films form barriers between the water and the solder. “In fact, just like lead paint ages and creates hazards as it degrades, so too does lead solder,” Edwards says. The presence of particles changes the importance of drinking water as a lead source, says Ralph Scott, community projects director of the Alliance for Healthy Homes, an advocacy group. “In most cases we have assumed and still assume that water is a small part of the problem compared to paint,” he says. “But now we are looking at a dimension of the water problem never considered before.”

Lead, covered by the 1991 Lead and Copper Rule, is one of the few drinking water contaminants monitored at the household tap. However, Edwards says that current regulations may miss tap water lead for a number of reasons. These include the current testing method, which he believes does not adequately measure particulate lead. Also, regulations require 90% of tested dwellings to meet the regulatory requirements but say nothing about the upper 10% of dwellings, which can have very high lead levels. Edwards and colleagues described these concerns in the June 2007 *Journal of the American Water Works Association*.

But EPA spokesperson Sakeba Carter-Jenkins says the agency has seen no data indicating its current methods miss particulate lead. “If EPA does receive data showing that these procedures need modification, it can be considered as part of the long-term revisions to the Lead and Copper Rule,” says Carter-Jenkins.

The EPA plans to begin reviewing the Lead and Copper Rule next year. Last year, the agency changed the rule so that water providers must report to the state before changing their water treatment regimens. This will allow for more timely monitoring of any changes in water quality at the tap, says Martha Keating, outreach coordinator with the Children’s Environmental Health Initiative at Duke University.

